# Evolution of Microstructure in Welding Heat-Affected Zone of G115 Steel with the Different Content of Boron

**DOI:** 10.3390/ma15062053

**Published:** 2022-03-10

**Authors:** Zhongyi Chen, Dongxu Kou, Zhengzong Chen, Fan Yang, Yonglin Ma, Yiming Li

**Affiliations:** 1 School of Material and Metallurgy, Inner Mongolia University of Science and Technology, Baotou 014010, China; kdx2020023011@163.com (D.K.); malin@imust.cn (Y.M.); 2 Institute for Special Steels, China Iron and Steel Research Institute, Haidian, Beijing 100081, China; czz1223@126.com; 3 Inner Mongolia Shangdu Power Company, Xilin Gol League, Xilinhot 027200, China; yfan202202@163.com; 4 Key Laboratory of Advanced Metals and Materials, School of Materials and Metallurgy, Inner Mongolia University of Science and Technology, Baotou 014010, China; liyiming79@sina.com

**Keywords:** G115 steel, heat-affected zone, M_23_C_6_ carbide, boron, phase transformation

## Abstract

Welding thermal simulation was performed to investigate the effects of boron content (0, 60, and 130 ppm), welding peak temperature (*T*_p_), and cooling time from 800 to 500 °C (*t*_8/5_) on the microstructure, carbide, subgrain, and microhardness of heat-affected zone (HAZ) in G115 steel. According to the experimental results, the microstructure of coarse-grained HAZ (CGHAZ), fine-grained HAZ (FGHAZ), inter-critical HAZ (ICHAZ), and sub-critically HAZ (SCHAZ) was martensite, martensite containing a small amount of undissolved carbide, martensite, and over-tempered martensite, tempered martensite, respectively. The presence of B element improved the thermal stability of M_23_C_6_ carbide, thereby resulting in a greater amount of undissolved carbides with a larger diameter in the materials with higher B content under the same *T*_p_. Element B is effective in improving *A*_c1_ and *A*_c3_ for the material. Besides, compared with the material without and containing 60 ppm B, the *A*_c1_ and *A*_c3_ of the material containing 130 ppm B increased by 95 and 108 °C, 69 and 77 °C, respectively. Meanwhile, the FGHAZ area of the material containing 130 ppm B was significantly lower than the material without or containing 60 ppm B, indicating that element B can significantly reduce the formation range of FGHAZ. The alloy content in austenite of ICHAZ of materials without or containing 60 ppm B increased, compared with CGHAZ, its *M*_s_ and *M*_f_ declined by 50 and 7 °C, 46 and 7 °C, respectively. In contrast, the alloy content in austenite of the material with 130 ppm B content decreases, its *M*_s_ and *M*_f_ was 37 °C and 32 °C higher than CGHAZ, respectively. The microhardness of HAZ was ranked in descending order as CGHAZ, FGHAZ, ICHAZ, and SCHAZ. Differently, the microhardness of CGHAZ and FGHAZ showed an increasing trend with the rise of B content but exhibited a decreasing trend with the rise of *t*_8/5_.

## 1. Introduction

Compared with the costly nickel-based superalloys, 9%Cr martensitic heat-resistant steel has been widely applied in various settings due to its better cost-effectiveness and practicability. Among them, P/T91 and P/T92 are the typical materials used in ultra-supercritical power generating units, the upper limit of service temperature of which is roughly 628 °C [1,2]. The improvement to steam parameters of thermal power generating units enhances the efficiency of power generation. It can also reduce coal consumption and carbon emissions, which have been burgeoning in many countries around the world. Among them, the new heat-resistant steel used in 630–650 °C ultra-supercritical generating units is the priority for development currently [3,4]. Developed by China Iron & Steel Research Institute Group (CISRI), G115 steel represents a new-generation ultra-supercritical martensitic heat-resistant steel, whose service temperature can be increased to as high as 650 °C [5]. Yan et al. [6,7,8] conducted a study on the effects of alloy content, hot working process, and heat treatment system on the microstructure and properties of G115 steel. Xiao et al. [5,9] explored the mechanism of long-term creep microstructure degradation and the creep physical yield strength model of G115 steel. Liu et al. [10] investigated the effect of B content (0, 60, 140 ppm) on the microstructure and properties of G115 steel after long-time aging at 650 °C. Properly increasing B content would reduce the coarsening rate of M_23_C_6_ carbides slowing down the reduction rate of dislocation density. Mechanical properties of G115 steels containing B of 140 ppm after aging were optimal. Moreover, Liu et al. [11] also studied the effect of W content (2.3, 2.6, 3.0 wt.%) on the microstructure and properties of G115 steel after aging, the coarsening rates of the martensitic lath and Laves phase increased with the increase in W content during aging. The G115 steel containing W of 2.3 wt.% revealed the best creep property during service in the three steels. Based on this, Liu et al. [12] continued to report the effect of W content (2.3, 2.6, 3.0 wt.%) on the creep properties of G115 steel at 650 °C. It was concluded that the coarsening rate of the lath and Laves phase were found to increase with the W content. The materials containing 2.3 wt.% W had the longest creep fracture life. The above conclusions are consistent with the literature [11]. Jing et al. [13] investigated the low-cycle fatigue behavior and microstructure evolution of G115 steel at 650 °C.

Since HAZ, as formed during welding in traditional 9%Cr martensitic heat-resistant steel, is significantly different from the base metal in mechanical properties, it usually represents the weak zone of welded joints. The *T*_p_ of CGHAZ exceeds 1300 °C, its microstructure is observed as coarse lath martensite, while the microstructure and properties exhibited by CGHAZ after post-weld heat treatment (PWHT) are comparable to those of the base metal [14]. Guo et al. [14] determined the microstructure and properties of MarBN steel (9Cr–3W–3Co steel) in HAZ welded state and PWHT by using the thermal simulation method. The results showed that HAZ had an obvious change in grain size. After PWHT, CGHAZ had a better distribution of precipitation phases, and its properties were optimal, and ICHAZ found a considerable Laves phase and had the worst properties. The *T*_p_ in FGHAZ is slightly over *A*_c3_, and the microstructure is observed as fine-grained martensites with a tiny amount of undissolved carbides. Within this sub-zone, the minimum creep strain rate of the material shows an increasing trend with the extension of service time, which reduces the service time and makes the material prone to type IV failure [15,16,17,18,19]. Through a HAZ high-temperature creep crack growth test conducted on P92 steel, Zhao et al. [19] discovered that creep crack behavior varied from one sub-zone of welded joints to another, with FGHAZ showing the maximum rate of creep crack growth. Abe et al. [15,16], Albert et al. [20], Liu et al. [21], and Wang et al. [22] conducted a study on the microstructure and properties of HAZ of 9%Cr martensitic heat-resistant steel, which led to the finding that the type IV failure suffered by FGHAZ was largely attributable to the complex stress state, small grain size, the uneven distribution of carbides, the reduction in the number of subgrains, and the formation of coarse Laves phase. Therefore, one of the effective solutions to preventing type IV failure is to compress FGHAZ fine-grained martensite. As the *T*_p_ of ICHAZ ranges between *A*_c1_ and *A*_c3_, the microstructures are dominated by martensite and over-tempered martensite. However, its mechanical properties are rendered poor by the significant difference in microstructure and properties between the martensite and over-tempered martensite. Consequently, it is also the sub-zone where HAZ is susceptible to the deterioration of property at high temperatures. During a study on the high-temperature creep microstructure and properties of HAZ of P92 steel, Ardghail et al. [23], An et al. [24] found out that the mixed microstructure of ICHAZ was the most likely site where a creep cavity can develop in the high-temperature creep process, which can result in the premature failure occurring to the material. Although *T*_p_ of SCHAZ is lower than that of *A*_c1_, it will still lead to the coarsening of carbides, the decrease of dislocation density, and the number of subgrains, thereby causing the mechanical properties to decline [14]. Trace element B can cause a significant impact on the mechanical properties of G115 steel at high temperatures. By exploring the microstructure and properties of G115 steel after long-term aging and high-temperature creep, Liu et al. [10] discovered that boron atoms could concentrate on the surface of M_23_C_6_ to replace part of carbon atoms in the material containing 140 ppm B, which is conducive to improving not only the thermal stability of carbides but also the creep properties of the material. Through a study carried out on the high-temperature mechanical properties and microstructure exhibited by the welded joints of 9Cr–3W–3Co heat-resistant steel with varying B content, Abe et al. [16] revealed that the welded joints of the material with 100 ppm B content possessed the high-temperature creep properties comparable to the base metal, but with no obvious type IV failure observed. Abe et al. applied the theory of α to γ reverse transformation during heating in the base metal microstructure to account for the reduction in fine-grained martensite of FGHAZ and the effective prevention of type IV failure. To sum up, it is necessary to further explore whether the same mechanism applies to the positive role played by element B in base metal and welding HAZ. In this study, the effect of B content on the phase transformation, carbides, subgrains, and microhardness of G115 steel at different *T*_p_ and *t*_8/5_ was investigated using a welding thermal simulation. It was concluded that B element would increase *A*_c1_ and *A*_c3_ and decrease *M*_s_ and *M*_f_. Properly increasing B content could compress the FGHAZ region, which is beneficial to improving creep properties in HAZ. The effect of B element on the HAZ microstructure of G115 steel was obtained, which explains the reason for the positive function of B element in HAZ and improves our understanding of the improvement of mechanical properties for base metal and HAZ by element B at high temperatures.

## 2. Experimental Material and Method

**Figure 1:**
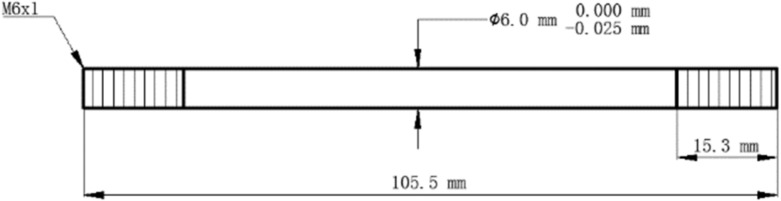
Schematic diagram of the sample.

As the experimental material, G115 steel consists of three different materials with varying B content (0, 60, and 130 ppm), whose chemical composition is detailed in Table 1. A small amount of powder was extracted from experimental material, and the content of C element in the steel was determined by infrared sulfur and carbon analyzer, the model and analytical accuracy of which are CS230 and RSD ≤ 0.5%, respectively. The content of N element in the steel was determined by O, N, and H analyzer, and its model and analytical accuracy are ONH836 and RSD < 2%, respectively. The rest of the elements (Si, Mn, Cr, Co, W, V, Nb, Cu, B) were analyzed by an inductively coupled plasma emission spectrometer. The corresponding model and analytical accuracies are Optima 8300DV and RSD ≤ 1.5%. Firstly, a vacuum electric arc furnace was employed for smelting and casting. After being molded, the steel ingot was austenitized at 1150–1210 °C and then hot forged into *ϕ* 16 mm test bars. Secondly, the steel ingot was normalized and held for 1.5 h (air cooling) at 1080 °C, and then backfired and held for 3 h (air cooling) at 775 °C. Following heat treatment, the test bar was cut into multiple thermal simulation samples with a size of *ϕ*6 × 105.5 mm. The schematic diagram of the sample is shown in Figure 1. Thirdly, the welding thermal simulation test was carried out on the Gleeble-1500D thermal simulator, the experimental plan of which is presented in Table 2. The Gleebe-1500D thermal simulator, an ideal physically simulated HAZ testing machine whose heating speed range of 0.002–1000 °C/s and a cooling speed range of 0.002–140 °C/s, is used to perform welding thermal simulation experiments. The temperature-dilatometric curve can be obtained by measuring the radial expansion in the homogeneous temperature zone through the radial dilatometer during the specimen’s heating, holding, and cooling. After that, *A*_c1_, *A*_c3_, *M*_s,_ and *M*_f_ are obtained by the tangent method. Fourthly, the room temperature was raised to 150 °C at a heating rate of 20 °C/s and maintained for 300 s before being further increased to the *T*_p_ at a heating rate of 200 °C/s and held for 1 s. Lastly, it was cooled down to room temperature at a predetermined *t*_8/5_, thus completing the thermal cycle simulation process. In the experiment, there were a total of four *T*_p_, which are 1350, 1150, 950, and 770 °C, corresponding to CGHAZ, FGHAZ, ICHAZ, and SCHAZ, respectively. Besides, there were a total of three welding *t*_8/5_ under each *T*_p_, which were 25, 50, and 150 s, respectively. According to the dilatometric curve, *A*_c1_, *A*_c3_, *M*_s_ and *M*_f_ under different *t*_8/5_ were determined. With the mixed solution of FeCl_3_ (10 g), HCl (30 mL) and H_2_O (160 mL) used to corrode the polishing surface of the sample for 30 s, the microstructure was observed with the assistance of a scanning electron microscope (SEM, Super55). The thin films were prepared using double-jet polishing in the mixed solution150 mL HClO_4_ and 850 mL CH_3_CH_2_OH, with the temperature set to 25 °C and the voltage set to 30 V. The microstructure and carbides were observed using the TEM (JEM-2100). Subsequently, the Vickers hardness tester (W-20) was applied to measure the microhardness of the samples, with a load of 10 N applied for 30 s. Each sample was tested three times, with the average taken. The mean diameter of original austenite grains in each sample was obtained using the linear intercept method on the metallographic images.

## 3. Results

### 3.1. Microstructure of Base Metal

**Figure 2:**
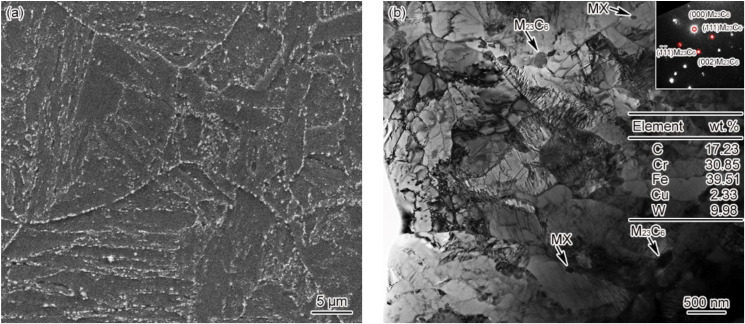
Microstructure of base metal (**a**) 3#–SEM, (**b**) 3#–TEM.

Figure 2 shows the microstructure of the base metal of G115 steel, which is identified as tempered martensite. As the main precipitates of G115 steel, the round Cr/Fe/W-rich M_23_C_6_ carbide with a diameter of 90–140 nm and the round V/Nb-rich MX carbonitride with a diameter of 50–70 nm constitute the main strengthening phases that play a vital role in maintaining the high-temperature creep properties of the material. When G115 is put into service for a long time, M_23_C_6_ will be coarsened, and the enhancement effect will diminish, with the coarsening rate of MX reaching a significantly lower level compared to M_23_C_6_ [9,13]. As a result, the carbide present in G115 steel will continue changing as the service is extended. As another form of strengthening mechanism, dislocation strengthening is not comparable only to solid solution strengthening and precipitation strengthening. Not only is the dislocation density in G115 steel extremely high, but there is also a prevalence of dislocation entanglement and subgrains on the slats, as shown in Figure 2b. In fact, this ensures the excellent creep properties of the material at high temperatures. With the service time extension, there would be a progressive decline in the dislocation density and the number of subgrains. There is barely any variation in microstructure and grain size for the base metal of materials 1–3#, while the average grain size falls into the range of 46 ± 4 μm.

### 3.2. Dilatometric Curve and Microstructure of CGHAZ

**Figure 3:**
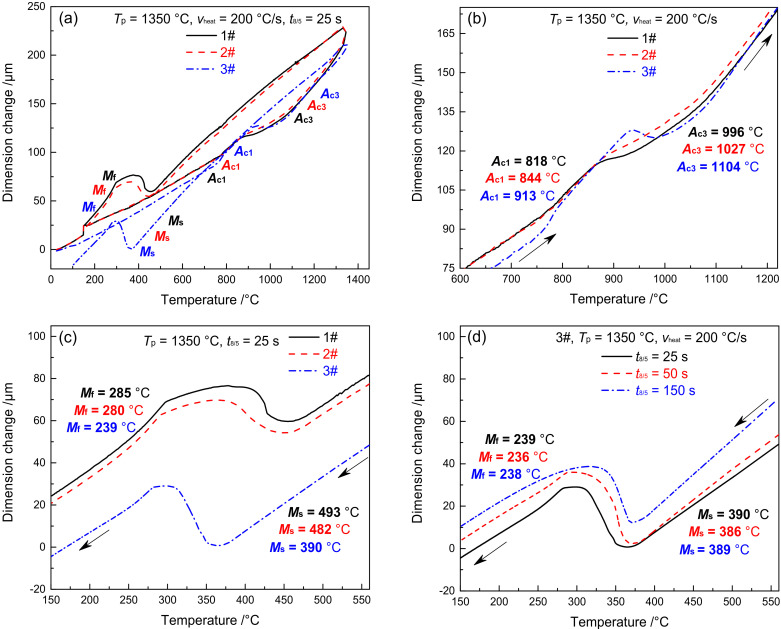
Dilatometric curves in CGHAZ. (**a**) complete dilatometric curves, (**b**) heating process, (**c**) cooling process, (**d**) 3#–the cooling process under different *t*_8/5_.

Figure 3 shows the dilatometric curves of CGHAZ of materials 1–3#. Figure 3a presents the complete dilatometric curves for materials 1–3# when t_8/5_ is 25 s. Figure 3b shows the partial dilatometric curves for materials 1–3# during the heating process. Figure 3c presents the partial dilatometric curves for materials 1–3# during the cooling process. Figure 3d shows the local dilatometric curves for material 3# during the cooling process given varying *t*_8/5_. As shown in Figure 3a, when the heating rate was 200 °C/s and *T*_p_ was 1350 °C, the base metal (tempered martensite, M_Tempered_) was completely transformed into austenite, which preceded the change of supercooled austenite into martensite. During the heating and cooling process of welding, the variation in B content between materials 1–3# led to an evident difference not only in *A*_c1_ and *A*_c3_ but also in *M*_s_ and *M*_f_. The *A*_c1_ and *A*_c3_ of materials 1–3# are 818 and 996 °C, 844 and 1027 °C, and 913 and 1104 °C, respectively, as shown in Figure 3b. The *A*_c1_ and *A*_c3_ of material 3# are 95 and 108 °C higher than material 1#, while they are 69 and 77 °C higher than material 2#. The *M*_s_ and *M*_f_ of materials 1–3# are 493 and 285 °C, 482 and 280 °C, and 390 and 239 °C, respectively. As shown in Figure 3c, the *M*_s_ and *M*_f_ of material 3# are 103 and 46 °C lower compared to material 1#, and they are 92 and 41 °C lower compared to material 2#. To sum up, element B is effective in increasing the *A*_c1_ and *A*_c3_ of the material and reducing the *M*_s_ and *M*_f_, with an increasing trend shown with the rise of B content. As shown in Figure 3d, the *M*_s_ and *M*_f_ of material 3# are maintained at 389 and 238 °C, respectively. This shows that the change of *t*_8/5_ will not affect *M*_s_ and *M*_f_ of material 3#.

**Figure 4:**
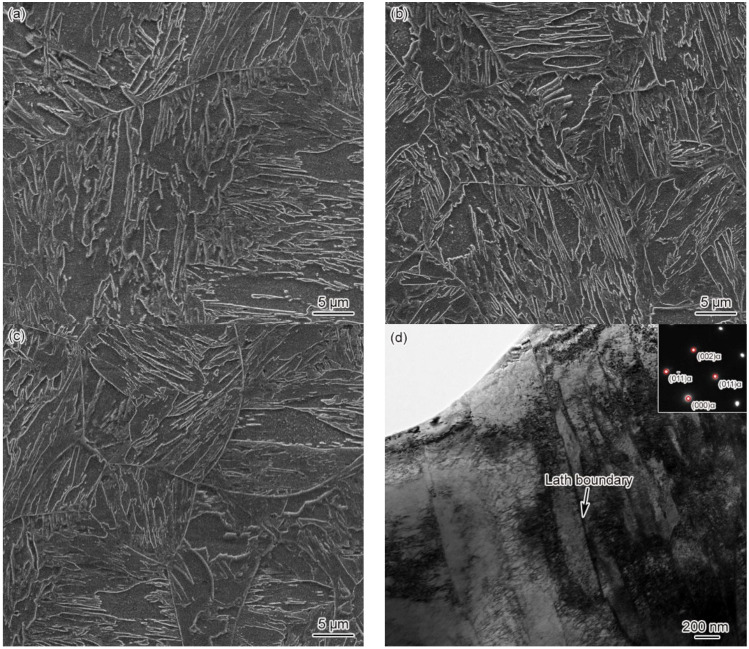
Microstructure of CGHAZ, *t*_8/5_ = 25 s (**a**) 1#–SEM, (**b**) 2#–SEM, (**c**) 3#–SEM, (**d**) 3#–TEM.

Figure 4 shows the microstructure of CGHAZ in materials 1–3#. It can be seen clearly from Figure 4 that the CGHAZ microstructure of G115 steel is typical coarse lath martensite. By comparing CGHAZ microstructure between materials 1–3#, it can be seen that the change in B content makes no difference to CGHAZ microstructure, as shown in Figure 4a–c. The average grain size of CGHAZ is 67 ± 3, 63 ± 3, 54 ± 4 μm, respectively, all of which are larger compared to the base metal (46 ± 4 μm), indicating a negative correlation between average grain size and B content. Since the *T*_p_ (*T*_p_ = 1350 °C) of CGHAZ is substantially higher than that of *A*_c3_, the carbides present in the base metal are completely dissolved into the matrix, while the austenite grain boundaries cannot be pinned effectively. Besides, with the austenite grains developing to the full, the final grain size becomes significantly larger compared to the base metal. Element B can affect the thermodynamics and dissolution behavior of carbides, thus making a difference to the pinning effect on grain boundaries and austenite grain size. The above shows that the grain size of material 3# is smaller relative to materials 1 and 2#, indicating that element B can indirectly affect the grain size. According to TEM observation on the CGHAZ of material 3#, the microstructure of CGHAZ was lath martensite, with the prevalence of dislocation tangle and subgrains in the lath. However, no undissolved carbides were found, as shown in Figure 4d.

According to Figure 3; Figure 4 in combination, the law of CGHAZ phase transformation of G115 steel can be visualized as follows:

$$M_{\mathrm{Tempered}}\overset{\mathrm{heating}\;(1350\;{}^{\circ}C)}{\to }\gamma \overset{\mathrm{cooling}}{\to }M$$

### 3.3. Dilatometric Curve and Microstructure of FGHAZ

**Figure 5:**
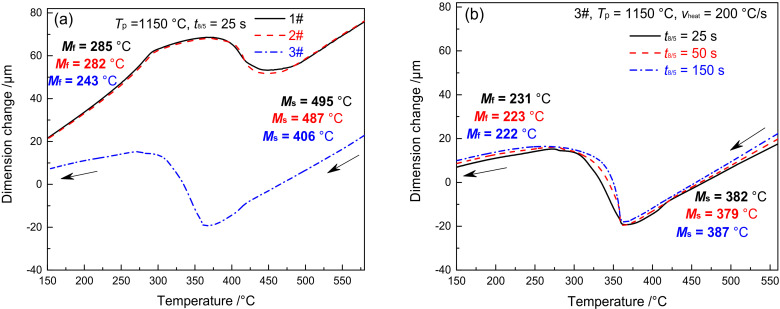
Dilatometric curves in FGHAZ (**a**) cooling process, (**b**) 3#–cooling process under different *t*_8/5_.

Figure 5 shows the dilatometric curves of FGHAZ for materials 1–3#. Except for *T*_p_, the dilatometric curve of FGHAZ is consistent with that of CGHAZ, while the microstructure of the base metal shifts to austenite and then to martensite, as shown in Figure 5a. The *M*_s_ and *M*_f_ of materials 1–3# are 495 and 285 °C, 487 and 282 °C, 406 and 243 °C, respectively, which are slightly higher than CGHAZ. The higher the B content, the higher the *M*_s_ and *M*_f_. Although the *T*_p_ of FGHAZ is higher than that of *A*_c3_, there remain a tiny amount of undissolved carbides. In addition, the alloy content and stability in austenite decrease slightly compared with CGHAZ, thereby leading to a slight improvement of *M*_s_ and *M*_f_. As shown in Figure 5b, the *M*_s_ and *M*_f_ of material 3# show no significant change given varying *t*_8/5_, suggesting that *t*_8/5_ makes little difference to the *M*_s_ and *M*_f_ of FGHAZ in G115 steel.

**Figure 6:**
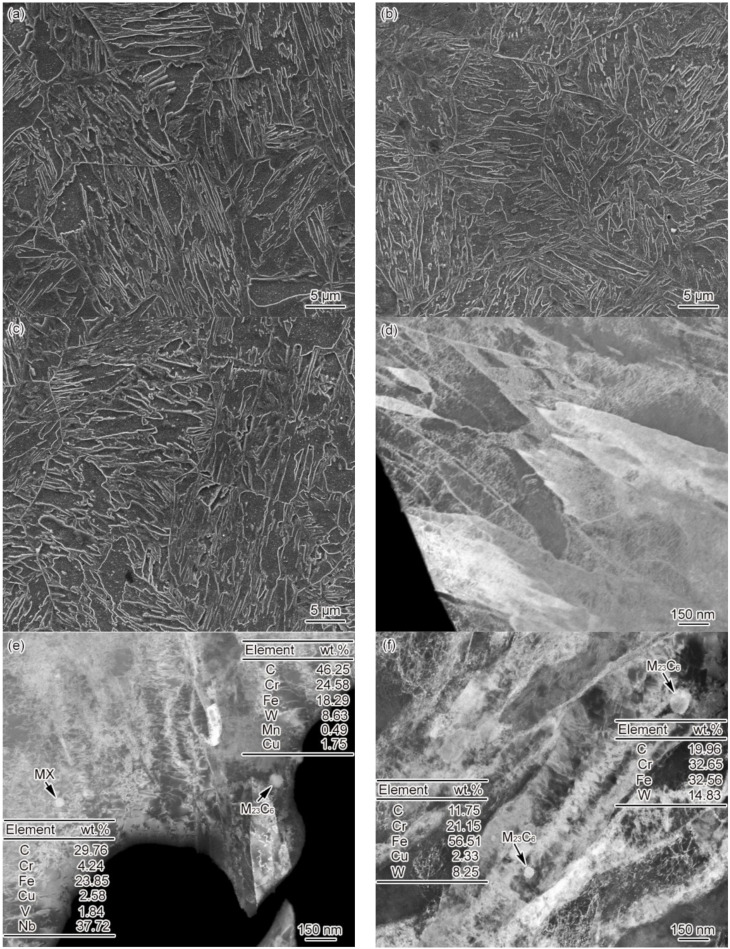
Microstructure of FGHAZ, *t*_8/5_ = 25 s (**a**) 1#–SEM, (**b**) 2#–SEM, (**c**) 3#–SEM, (**d**) 1#–TEM, (**e**) 2#–TEM, (**f**) 3#–TEM.

Figure 6 shows the FGHAZ microstructure of materials 1–3# at *t*_8/5_ of 25 s. The FGHAZ microstructure of materials 1–3# is fine lath martensite, with no significant differences observed between these three materials, as shown in Figure 6a–c. As revealed by the SEM images, there was no presence of undissolved carbides in the microstructure. The average grain size of FGHAZ original austenite falls into the range of 32 ± 3 and 26 ± 2, 21 ± 4 μm, respectively. The larger the B content, the smaller the grain size. In addition, it can be seen from Figure 6d–f that the FGHAZ microstructure of materials 1–3# is lath martensite. Besides, the lath shows not only a high density of dislocation but also dislocation tangle and subgrains to some extent. However, no undissolved carbides were observed in material 1#, despite a small amount of circular undissolved M_23_C_6_ and MX discovered inside the lath of materials 2 and 3#. According to the statistical results, the diameters of undissolved carbides in materials 2# were determined at about 67–78 nm, the diameter of MX was determined at about 29–38 nm, and the diameter of undissolved M_23_C_6_ in material 3# was determined at about 51–96 nm. The diameter and amount of undissolved carbides in FGHAZ were smaller than the base metal, while those of undissolved carbides in material 3# were slightly larger than in material 2#. These results demonstrate that the B content can impact the thermal stability of FGHAZ carbides in G115 steel, which leads to completely different distribution patterns for undissolved carbides.

The undissolved M_23_C_6_ mostly appears in the martensitic lath, indicating that M_23_C_6_ in the base metal has no time to redissolve during the heating process, is surrounded by austenite, and finally remains in the martensitic lath. Differently, more of the M_23_C_6_ present in the base metal emerged on the grain boundary and the lath boundary, with significant variations shown in location, amount, and size. By comparing the average grain size and undissolved carbides of FGHAZ of materials 1–3#, it can be found that B content affected the stability of carbides [10], thus making a difference to the austenitizing process and the grain size of these three materials. Meanwhile, the *A*_c1_ and *A*_c3_ of materials 1–3# varied due to the difference in B content. When *T*_p_ = 1150 °C, the *A*_c3_ of material 1# is 996 °C, and its *T*_p_ is higher than *A*_c3_, reaching 154 °C, which is sufficient to ensure that the carbides in the base metal can be completely redissolved. For material 2#, its *A*_c3_ is 1027 °C, and *T*_p_ is 123 °C higher than *A*_c3_. This is also enough for most of the carbides in the base metal to be redissolved, despite a small number of undissolved carbides that remain. For material 3#, its *A*_c3_ is 1104 °C, and *T*_p_ is merely 46 °C higher than *A*_c3_. In this case, part of the carbides in the base metal can be redissolved, and some undissolved carbides remain. The amount of undissolved carbides is inversely correlated with the difference between *T*_p_ and *A*_c3_.

According to Figure 5; Figure 6 in combination, the FGHAZ phase transformation rule of G115 steel is expressed as follows:

$$M_{\mathrm{Tempered}}\overset{\mathrm{heating}\;(1150\;\text{°}C)}{\to }\gamma \overset{\mathrm{cooling}}{\to }M$$

### 3.4. Dilatometric Curve and Microstructure of ICHAZ

**Figure 7:**
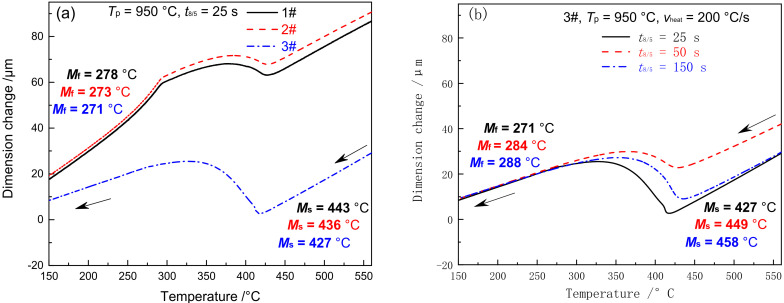
Dilatometric curves in ICHAZ (**a**) cooling process, (**b**) 3#–the cooling process under different *t*_8/5_.

Figure 7 shows the dilatometric curves of ICHAZ in materials 1–3#. With the *T*_p_ of ICHAZ ranging between *A*_c1_ and *A*_c3_, the base metal was partially transformed into austenite and then into martensite during the subsequent cooling process. The parts left not transformed were over-tempered martensite [25]. Due to the difference in austenite content and alloy content in the austenite formed by materials 1–3# at *T*_p_ (*T*_p_ = 950 °C), the cooling transition curve differed to CGHAZ. The *M*_s_ and *M*_f_ of materials 1 and 2# were 443 and 278 °C, 436 and 273 °C, respectively, which are 50 and 7 °C and 46 and 7 °C lower compared to CGHAZ, respectively. The *M*_s_ and *M*_f_ of material 3# was 427 °C and 271 °C, respectively, which is 37 °C and 32 °C higher compared to CGHAZ. When *t*_8/5_ reached 25, 50, and 150 s, respectively, the *M*_s_ of material 3# was 427, 449 and 458 °C respectively, while the *M*_f_ was 271, 284, and 288 °C respectively, as shown in Figure 7b. It is indicated that both *M*_s_ and *M*_f_ rose slightly with the increase of *t*_8/5_. The above changes in *M*_s_ and *M*_f_ for materials 1–3# were closely associated with the amount of austenite formed and that of carbides redissolved at *T*_p_.

**Figure 8:**
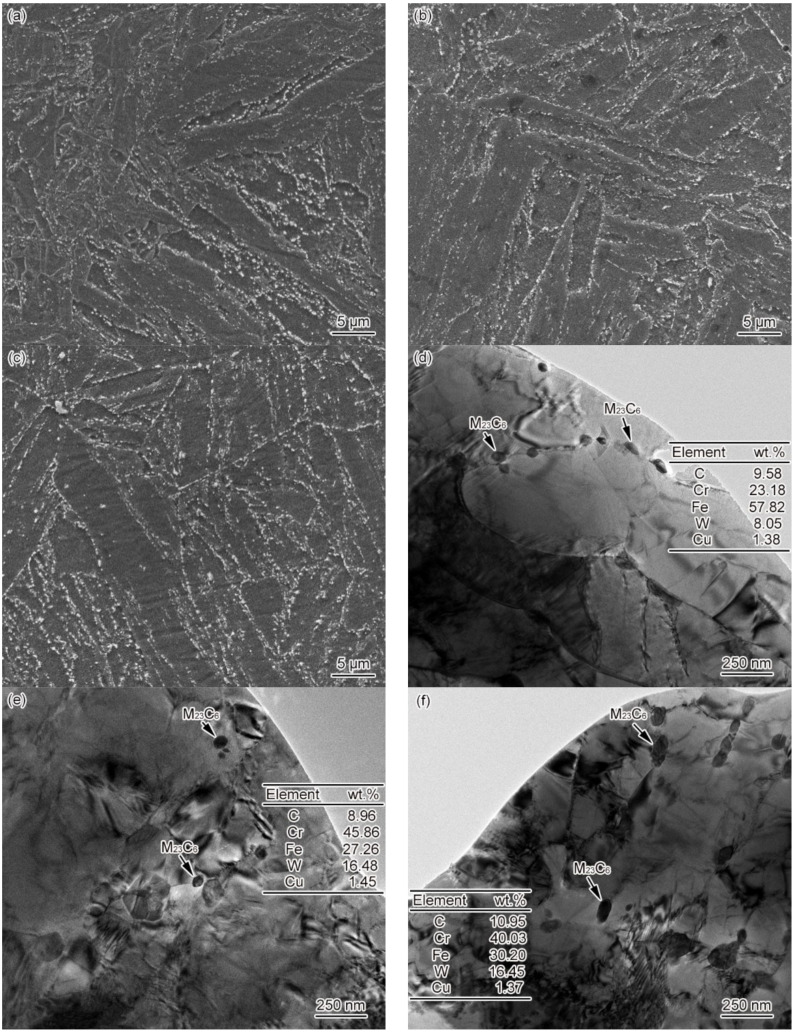
Microstructure of ICHAZ, *t*_8/5_ = 25 s (**a**) 1#–SEM, (**b**) 2#–SEM, (**c**) 3#–SEM, (**d**) 1#–TEM, (**e**) 2#–TEM, (**f**) 3#–TEM.

Figure 8 shows the microstructure of ICHAZ in materials 1–3#. ICHAZ microstructure presents itself as a mixture of martensite and over-tempered martensite. Less tiny carbides were distributed in the martensite region, while the grain boundary and lath boundary of original austenite were blurred in the over-tempered martensite region. Besides, there were plenty of carbides of different sizes that were unevenly distributed, as shown in Figure 8a–c. Due to the variation in the austenite content as formed by materials 1–3# at *T*_p_, the content of martensite as formed by material 1# was the highest, followed by materials 2 and 3#. According to Figure 8d–f, there was a large amount of M_23_C_6_ present in the ICHAZ microstructure. The diameter of M_23_C_6_ ranges from 47 to 73 nm, which is significantly smaller than the carbide size of the base metal, indicating the ill-timed redissolution of M_23_C_6_ in the heating process of welding. Consequently, undissolved M_23_C_6_ resulted. The diameter of material 2# falls into the 52–79 nm range, with the same situation occurring. The diameter of material 3# ranges from 70 to 124 nm, with M_23_C_6_ concentrating in some particular regions. It can be seen from the above that the B content affected the thermal stability of M_23_C_6_, which makes a difference to the distribution of undissolved carbides in ICHAZ for materials 1–3#. The distribution of undissolved carbides showed similarity to that of FGHAZ, i.e., the number of undissolved carbides was inversely correlated with the difference between *T*_p_ and *A*_c1_. The difference between *T*_p_ and *A*_c1_ of 1# material was most significant, while the amount and size of undissolved carbides were the smallest. Similarly, given the least significant difference between *T*_p_ and *A*_c1_ of material 3#, the amount and size of undissolved carbides were the highest.

According to Figure 7; Figure 8 in combination, the law of ICHAZ phase transformation of G115 steel can be expressed as follows:

$$M_{\mathrm{Tempered}}\overset{\mathrm{heating}}{\to }{\gamma +M}_{\mathrm{Over}-\mathrm{tempered}}\overset{\mathrm{cooling}}{\to }{M+M}_{\mathrm{Over}-\mathrm{tempered}}$$

### 3.5. Dilatometric Curve and Microstructure of SCHAZ

**Figure 9:**
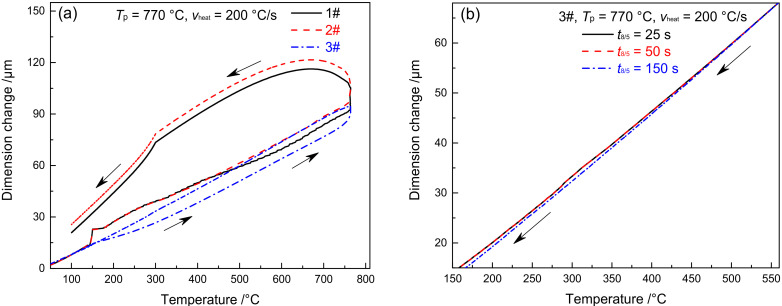
Dilatometric curves in SCHAZ (**a**) complete dilatometric curves, (**b**) 3#–the cooling process under different *t*_8/5_.

**Figure 10:**
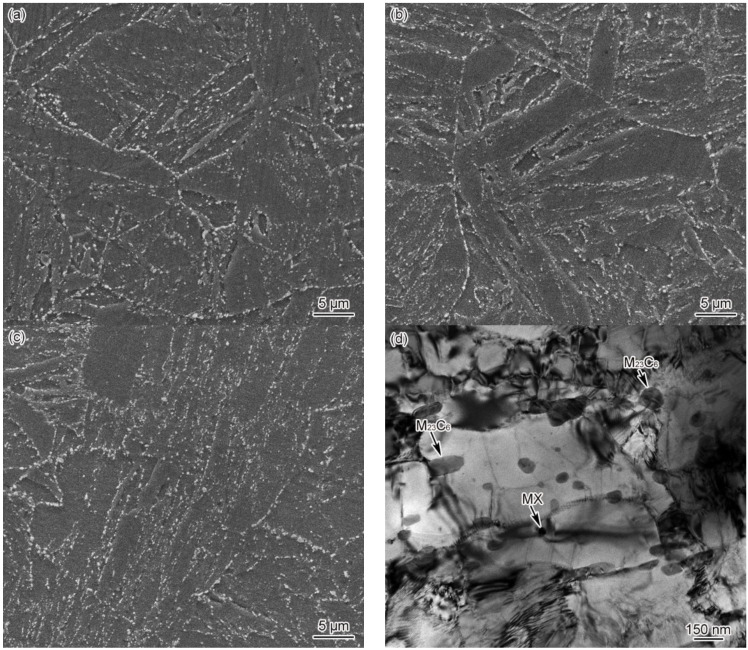
Microstructure of SCHAZ, *t*_8/5_ = 25 s (**a**) 1#–SEM, (**b**) 2#–SEM, (**c**) 3#–SEM, (**d**) 3#–TEM.

Figure 9 shows the dilatometric curves of SCHAZ in materials 1–3#. Since the *T*_p_ of SCHAZ was lower compared to *A*_c1_, there was no occurrence of austenitic transformation in the microstructure of the base metal, nor was any new phase formed during welding heating and cooling, as shown in Figure 9a. Meanwhile, *t*_8/5_ had no impact on the dilatometric curve, as shown in Figure 9b. Due to the high heating rate and short residence time during the welding thermal cycle, the microstructure barely changed. The microstructure of SCHAZ in materials 1–3# is tempered martensite, which is consistent with that of the base metal. Besides, there is no significant difference among the three materials, as shown in Figure 10. Compared with the base metal, the carbide size of SCHAZ increased proportionately. In contrast, there was a reduction in the dislocation density and the number of subgrains, despite not to a large extent.

Combined with Figure 9; Figure 10, the SCHAZ phase transformation rule of G115 steel is as follows:

$$M_{\mathrm{Tempered}}\overset{\mathrm{heating}}{\to }M_{\mathrm{Tempered}}\overset{\mathrm{cooling}}{\to }M_{\mathrm{Tempered}}$$

### 3.6. Microhardness of HAZ

**Figure 11:**
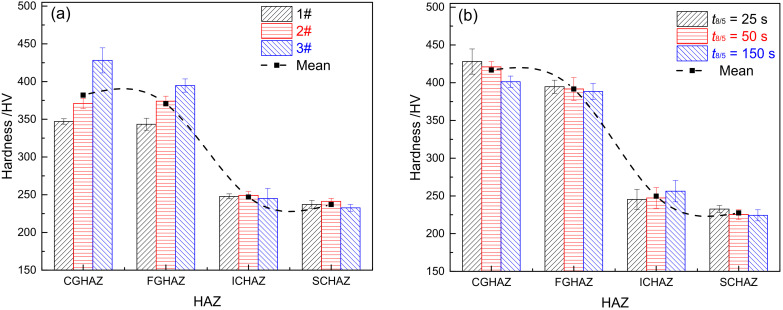
Microhardness of HAZ (**a**) materials 1–3# when *t*_8/5_ is 25 s, (**b**) material 3# under different *t*_8/5_.

Figure 11 shows the microhardness of HAZ in materials 1–3#. Figure 11a illustrates the distribution of HAZ microhardness in materials 1–3# when *t*_8/5_ is 25 s. From Figure 11a, it can be seen that the average microhardness of each sub-zone from high to low is CGHAZ (382 HV), FGHAZ (371 HV), ICHAZ (254 HV), and SCHAZ (237 HV). The trend of changes in HAZ microhardness is closely associated with the microstructure of each sub-zone. The microstructure is lath martensite for CGHAZ and FGHAZ, which are similar in microhardness. Besides, it is where the microhardness is the highest for each sub-zone. The microstructure of ICHAZ is over-tempered martensite and martensite, with the microhardness of ICHAZ being slightly higher than SCHAZ and base metal (232 HV). The microstructure of SCHAZ is tempered martensite, with the lowest microhardness reached. In CGHAZ and FGHAZ, the level of microhardness shows an upward trend with the increase of B content. Although the microstructure is lath martensite for CGHAZ and FGHAZ in materials 1–3#, there remains variation in their microhardness, which is directly related to the B content. Boron atoms can be adsorbed onto the supercooled austenite grain boundary, which can reduce grain boundary energy and the formation of common-lattice M_23_(CB)_6_ carbides, thereby significantly inhibiting the occurrence of austenite coarsening and high-temperature transformation. Ultimately, the hardenability and microhardness of materials are improved. In ICHAZ and SCHAZ, there is only an insignificant difference in the microhardness among materials 1–3#. With no complete austenitic transformation occurring to ICHAZ or SCHAZ, it is unlikely for element B to play its role as it does in CGHAZ and FGHAZ.

Figure 11b shows the microhardness distribution for HAZ in material 3# at varying *t*_8/5_. As revealed by Figure 11b, the trend of changes in HAZ microhardness for material 3# is consistent with that in Figure 11a. The average microhardness from high to low is CGHAZ (416 HV), FGHAZ (391 HV), ICHAZ (249 HV), and SCHAZ (227 HV). Except for ICHAZ, the microhardness in each sub-zone exhibits a declining trend with an increase of *t*_8/5_. The microhardness is related to the type of microstructure; it also relates to the grain size and the distribution of carbides. In CGHAZ and FGHAZ, the larger the *t*_8/5_, the larger the original austenite grain size, and the lower its microhardness [14]. Similarly, in SCHAZ, with the increase of *t*_8/5_, the residence time of the microstructure is extended at high temperatures, which leads to a further increased possibility of over-tempering for the material and causes further deterioration of the mechanical properties. In ICHAZ, the increase of *t*_8/5_ contributes to the formation of more austenites, which further increases the martensite content, thereby improving the level of microhardness.

## 4. Discussion

### 4.1. Evolution of Microstructure

To sum up, the microstructure of CGHAZ is coarse lath martensite. Following PWHT, tempered martensite will take shape, which is not only consistent with the microstructure and properties of the base metal. Besides, it represents the sub-zone with the best property in HAZ. The microstructure of FGHAZ is fine-grained martensite, where a small number of undissolved carbides exist. In addition, tempered martensite will also come into form in this sub-zone during PWHT. Due to the small size of grains and the presence of undissolved carbides; however, it is bound to make the properties different to CGHAZ. It is common sense that HAZ of P/T91 and P/T92 steels are prone to type Ⅳ failure, which is one of the main contributors to reducing their high temperature creep properties and service life [15,16]. The type Ⅳ failure is associated largely with the unique microstructure of FGHAZ [17]. It is easy for the undissolved carbides of FGHAZ to be coarsened in the fine-grained martensite during PWHT, which hinders the grain boundaries of fine-grained martensite from being pinned effectively. Meanwhile, undissolved carbides reduce the content of carbide-forming elements in original austenites. As a result, there are a large number of dispersed carbides that are unable to precipitate at the original austenite grain boundaries, lath boundaries of fine-grained martensites. Moreover, the fine-grained martensite with high diffusion channels plays a role in further accelerating carbide coarsening, which is adverse to improving the creep resistance at high temperatures. Currently, it has been confirmed that there are plenty of creep voids and coarse carbides existing on the grain boundaries of FGHAZ where type Ⅳ failure occurs [15,16]. Therefore, FGHAZ is considered the main sub-zone where type Ⅳ failure occurs to traditional martensitic heat-resistant steels given a lot of factors that could have adverse effects on high-temperature creep properties. In a study on the high-temperature creep properties exhibited by the welded joints of heat-resistant steels such as 9Cr–3W–3Co steel with an addition of 100 ppm B and MarBN steel with a composition similar to G115 steel, Abe et al. [15,16]. and Tabuchi et al. [18] discovered that the addition of boron effectively optimized the grain size of FGHAZ, with only a tiny amount of small grains surrounding coarse grains. Meanwhile, the variation in high-temperature creep property with the base metal was reduced, which is much better than the base metal of P/T91 and P/T92 steel, so that the likelihood of type Ⅳ failure was reduced.

The microstructure of ICHAZ is a mix of over-tempered martensite and martensite. Like FGHAZ, ICHAZ contains fine grained martensite, which could hinder the creep properties of materials from improving at high temperatures. With restoration occurring in most regions of over-tempered martensites, there would be a significant reduction in the dislocation density and the number of subgrains. At the same time, there is not sufficient time allowed for some carbides to be redissolved, thus resulting in the uneven distribution of different sizes, which is adverse to pinning grain boundaries. During PWHT, many undissolved carbides will be further coarsened, which is accompanied by a sharp decline in their high-temperature creep properties. As demonstrated by Guo et al. [14] in their study on the microstructure of HAZ, there would be a large number of Laves phases coming into form in ICHAZ after PWHT, and its properties would also deteriorate significantly. Through a study on the high-temperature creep microstructure and properties of HAZ of P92 steel, Ardghail et al. [23], An et al. [24] discovered that the mixed microstructure of ICHAZ had an adverse effect on the high-temperature creep properties of the material, and was the most likely sub-zone where a creep cavity takes shape in the high-temperature creep process, thus causing the occurrence of premature failure. The microstructure and properties of the martensite region are clearly different from those in the over-tempered martensite region, which affects the coordinated deformation mechanism during the deformation and creep process, thus increasing the severity of failure. The microstructure of SCHAZ remains tempered martensite, which is consistent with the base metal.

As shown in Figure 3, Figure 4, Figure 5, Figure 6, Figure 7, Figure 8, Figure 9 and Figure 10, CGHAZ, FGHAZ, ICHAZ, and SCHAZ exist in materials 1–3#, with a basic consistency shown in the microstructure of each sub-zone, indicating that the addition of B made no change to the sub-zone type and microstructure of HAZ in G115 steel. However, the addition of B did change the *A*_c1_ and *A*_c3_ of 1–3# materials, resulting in the different temperature ranges in their respective sub-zone and the different microstructure characteristics given the same *T*_p_. For example, the distribution state of undissolved carbides (size, quantity, shape, spacing) and grain size in materials 1–3# are different in FGHAZ. Such a slight change explains why the G115 steel containing 130 ppm B demonstrates excellent high-temperature creep property and the insensitivity to type Ⅳ failure.

### 4.2. Evolution of Carbides

Precipitation strengthening plays an important role in the strengthening mechanism for the heat-resistant steel containing 9%Cr [9,13]. Carbides are capable of pinning grain boundaries and free dislocations, which ensures the long-term microstructural stability of materials at the temperatures required for service. As shown in Figure 4, no undissolved carbides were observed in CGHAZ for materials 1–3#, indicating the complete dissolution of carbides. In this circumstance, the carbides consistent with the base metal could be re-formed in the PWHT process, as is their distribution. In FGHAZ, materials 1–3# showed variation in the distribution of undissolved carbide at the same *T*_p_ due to the difference in B content. Material 1# contained no undissolved carbides, while materials 2# and 3# did. The amount and size of carbides in material 3# were fairly large. As indicated by Abe et al. [15,16] in the analysis of type Ⅳ failure, the existence of undissolved carbides would affect the probability of type Ⅳ failure occurring. The undissolved carbides of FGHAZ are all contained the martensitic lath, as shown in Figure 6e,f. In the PWHT process, the undissolved carbides will be coarsened, and they cannot be effectively pinned for being absent from the original austenite grain or lath boundaries. The excellent high-temperature property of the base metal is directly related to the presence of carbides at the grain boundaries or the lath boundaries. Carbides are effective in pinning grain boundaries or lath boundaries, which ensures that there is no occurrence of grain boundary migration and lath coarsening at high temperatures [13]. However, this is adverse to the stability of the microstructure. Due to the existence of undissolved carbides, the content of carbon and alloying elements around them is low, which leads to reduced solution strengthening and dislocation strengthening in local areas, thus increasing the possibility of recovery. In general, the occurrence of the recovery signals the initiation of a decline in microstructure stability. Meanwhile, due to the existence of undissolved carbides and their coarsening in PWHT, the amount and size of re-precipitated carbides are significantly reduced, which further reduces the favorable factors for improving microstructure stability.

There are plenty of undissolved carbides in the martensites of ICHAZ. Due to the low *T*_p_ and short time of ICHAZ, the undissolved and coarsened carbides remain in their original positions, as shown in Figure 8d–f. As a result, the positive pinning effects on grain boundaries and lath boundaries remain in PWHT and service stages. However, the existence of the over-tempered martensite region will further promote the occurrence of restoration, thus accelerating the carbide coarsening and the formation of the Laves phase [14]. It is widely known that the formation of the Laves phase usually suggests the start of deterioration in the creep properties of materials at high temperatures. The carbides in SCHAZ showed no significant change.

**Figure 12:**
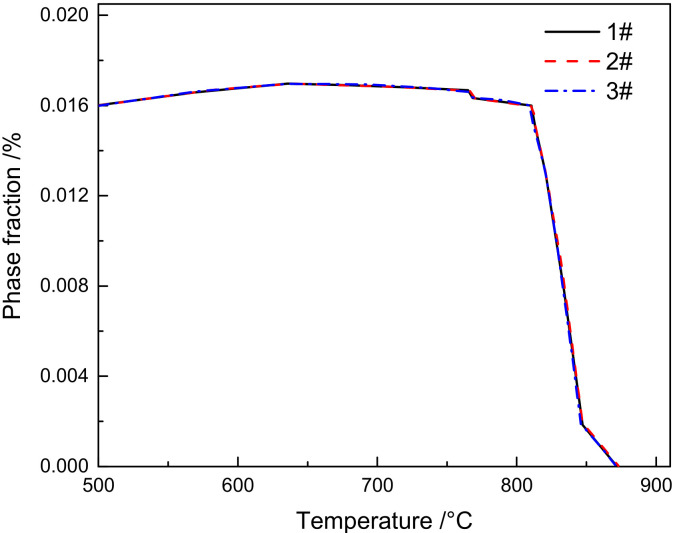
Influence of B content on M_23_C_6_ carbide.

Thermodynamic calculation on the equilibrium precipitated phase of M_23_C_6_ in 1–3# materials was carried out using the TCFe9 database in Thermo-Calc software. As seen in Figure 12, which shows the effect of B content on the amount of M_23_C_6_ carbide, we can find that the B content within the range of 0–0.013% has no obvious influence on the content of M_23_C_6_ carbide.

Liu et al. [10] studied the segregation content of B in M_23_C_6_ carbide in G115 steel containing 0, 60, and 140 ppm B content. The mixed powder of M_23_C_6_, Laves phase, and MX phase was prepared by the electrolytic method, after which the Laves phase and MX phase in the mixed powder were dissolved in 6% H_2_SO_4_ + 20% H_2_O_2_ + 2% citric acid + 72% H_2_O solution to obtain a single M_23_C_6_ powder. Finally, the M_23_C_6_ powder was dissolved in concentrated HNO_3_ and made a clear solution, and the content of each element in the solution was determined by spectrophotometry. Table 3 shows the structural formula of the composition of M_23_C_6_ in the three materials. As shown from Table 3, the B content in M_23_C_6_ increases with the increase of B content; meanwhile, the proportion of B atoms replacing C atoms also goes up.

The addition of element B caused a change to the thermodynamic stability of M_23_C_6_ carbide, thus affecting the distribution of M_23_C_6_. The boron atoms in G115 steel can replace part of the carbon atoms in M_23_C_6_, thereby giving rise to M_23_(CB)_6_. According to the literature [10], the content of B in M_23_C_6_ was about 0.56% when the content of B in G115 steel increased to 140 ppm, which indicates that boron atoms can replace carbon atoms in M_23_C_6_ and concentrate in its surface layer to which the degree increased with the rise in the amount of element B added. By applying the first principles to study the thermal stability mechanism followed for replacing some carbon atoms in M_23_C_6_ with boron atoms in 9Cr–3W–3Co steel, Sahara et al. [26] demonstrated that partial substitution of carbon atoms by boron atoms in M_23_C_6_ diminished the interfacial energy of carbides while improving their stability. Therefore, the thermal stability of M_23_C_6_ was low for material 1# when *T*_p_ reached 1150 °C, which is because it contained no element B and was completely dissolved at this temperature. However, since materials 2 and 3# contained element B, their M_23_(CB)_6_ possessed high thermal stability and failed to be fully redissolved at 1150 °C. This is a major contributor to the different distribution characteristics for the undissolved carbides in FGHAZ of materials 1–3#. Similarly, materials 1–3# also showed different characteristics of carbide distribution when *T*_p_ was 950 °C, that is, in the ICHAZ temperature range.

### 4.3. B’s Role in Compressing FGHAZ

As mentioned above, the welded joints of traditional 9%Cr martensitic heat-resistant steel are prone to type Ⅳ failure, which is closely related to the microstructure, grain size, carbide distribution, and stress state of HAZ. After an analysis of such heat-resistant steels as P91 and P92, Abe et al. [15,16] discovered that type IV failure usually occurred in FGHAZ, based on which the proposal was made to reduce the probability of type Ⅳ failure by compressing the formation of FGHAZ. By adding element B and reducing the content of N in 9Cr–3W–3Co steel, Abe et al. [15,16] obtained the creep properties of FGHAZ after compression through welding test. According to the results, the creep properties and durability of FGHAZ showed similarity to those of the base metal. On this basis, it was hypothesized that the tempered martensite completed the austenite transformation in reverse due to the addition of B, thereby explaining why the grain size of martensites of FGHAZ was close to the original austenite size of the base metal.

As shown in Figure 3a,b, the *A*_c1_ and *A*_c3_ of materials 1–3# are 818 and 996 °C, 844 and 1027 °C, and 913 and 1104 °C, respectively. The *T*_p_ of FGHAZ (*T*_p_ = 1150 °C) exceeds the *A*_c3_ of materials 1–3#. In combination with Figure 6, it can be seen that the microstructure of FGHAZ in materials 1–3# is lath martensite, with its average grain size reaching 32 ± 3, 26 ± 2, 21 ± 4 μm, respectively, which is significantly smaller compared to the base metal. It indicates the continued existence of FGHAZ. Guo et al. [12] reached the same conclusion about FGHAZ from the study on the microstructure of the MarBN heat-affected zone. Similarly, Abe et al. also observed a small number of fine-grained martensites during the study on the HAZ microstructure and properties of 9Cr–3W–3Co steel and MarBN steel. According to the above conclusion, FGHAZ remained in G115 steel containing element B. The high creep property and type Ⅳ failure resistance of G115 steel are directly related to the reduction in the area of FGHAZ. When the content of fine-grained martensite in HAZ is reduced, that is, the FGHAZ region decreases or is compressed, the type Ⅳ failure can be prevented to a certain extent, thus improving the creep properties.

Therefore, how was the FGHAZ region compressed? For the heat-resistant martensitic steel containing 9%Cr, *A*_c1_–*A*_c3_ is the range of ICHAZ formation temperature, and *A*_c3_–1200 °C or *A*_c3_–1250 °C is the range of FGHAZ formation temperature. In this experiment, when the welding heating rate is 200 °C/s, the residence time of materials 1–3# in the range of ICHAZ temperature is 0.89, 0.92, 0.96 s, respectively, which indicates only a slight difference between the three. When the range of formation temperature for FGHAZ is *A*_c3_–1200 °C, the range of FGHAZ formation temperature for materials 1–3# is 996–1200 °C, 1027–1200 °C, and 1104–1200 °C, respectively. While the residence time of materials 1–3# is 1.02, 0.87, and 0.48, respectively. The residence time of material 3# in FGHAZ decreased by 53.94% compared to material 1#. When the formation temperature range for FGHAZ is *A*_c3_–1250 °C, the residence time of materials 1–3# is 1.27, 1.12, and 0.73 s, respectively, and the residence time of material 3# in FGHAZ was reduced by 42.52% compared to material 1#. Therefore, it can be seen that 3# can effectively reduce the formation time of FGHAZ and thus compress its proportion and region, which is irrelevant to how the range of formation temperature for FGHAZ is defined.

The above phenomenon shows a direct correlation with the fact that the addition of B caused a change to the thermal stability of M_23_C_6_, thus increasing *A*_c1_ and *A*_c1_. Since the solubility of B in austenites is low (~0.018%), it is easier to be adsorbed into the defect regions such as grain boundaries and phase boundaries. When boron atoms concentrate along grain boundaries, both the grain boundary energy and the nucleation of austenites will be reduced, thereby causing delay to the austenitizing process [13,17,27]. Meanwhile, element B can reduce the austenite phase region, which is similar to the element in the closed austenite phase region, thus increasing *A*_c1_ and *A*_c3_. The boron atoms in G115 steel can replace part of carbon atoms in M_23_C_6_ carbide, thus resulting in the formation of M_23_(CB)_6_ [13,17,27]. The M_23_(CB)_6_ modified by element B showed improved stability and melting temperature, which is conducive to enhancing the pinning effects on the grain boundaries of austenites and inhibiting nucleation and growth. Thus, the process of austenitization was significantly delayed while *A*_c1_ and *A*_c3_ were significantly improved. Besides, the *A*_c1_ and *A*_c3_ increased with the increase of content B. The *A*_c3_ of material 2# is about 26 °C higher than material 1#, while the *A*_c3_ of 3# material is about 107 °C higher than material 1#. Based on this, the FGHAZ region formed in the welding process of G115 steel containing about 130 ppm B was small, which is effective in reducing the content of fine-grained martensites and improving the type Ⅳ failure resistance and creep property of the material.

**Figure 13:**
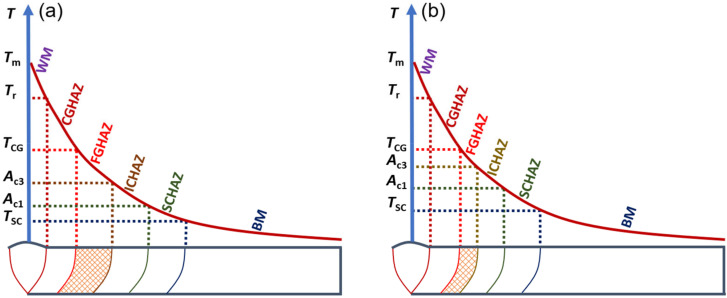
Influence of element B on FGHAZ area (**a**) B wt.% ≤ 60 ppm, (**b**) B wt.% = 130 ppm.

Figure 13 presents the schematic diagram of compression for FGHAZ in G115 steel by increasing the *A*_c3_. *T*_m_, *T*_r_, *T*_CG_, *T*_SC_ represent the weld joint peak temperature, the critical temperature of the welding fusion zone, the critical temperature of CGHAZ, and the critical temperature of SCHAZ, respectively. As shown in Figure 13, each subzone was taking shape in the HAZ of welded joints within a certain temperature range. The range of formation temperature for FGHAZ is *A*_c3_–*T*_CG_. When *T*_CG_ remained unchanged and *A*_c3_ increased, the formation temperature range for FGHAZ was compressed, and the area of FGHAZ was reduced. That is to say, the shadow area in Figure 13 was significantly reduced, and the FGHAZ martensite content declined, which is effective in inhibiting the occurrence of type Ⅳ failure.

### 4.4. Effects of B Content and t_8/5_ on M_s_ and M_f_

During the welding process, when the *T*_p_ exceeds *A*_c1_, the austenitic transformation will occur, thereby leading to the generation of phase transition products with a significantly different microstructure from the base metal. Due to the high alloy content in G115 steel and its excellent hardenability, the formed austenite will undergo martensite transformation in the cooling process. The effect of element B on the hardenability of materials is different from that of other alloying elements in austenite [28,29,30]. In the supercooled austenite of CGHAZ, the boron atoms adsorbed onto the grain boundaries can not only reduce the grain boundary energy but also give rise to common-lattice M_23_(CB)_6_, which can significantly inhibit the nucleation of the high-temperature phase (α-ferrite), extend the incubation period of austenite decomposition, enhance the stability of austenite, and thereby reduce the *M*_s_ and *M*_f_ to a significant extent. Due to the difference in B content between materials 1–3#, there is inevitably a difference in *M*_s_ and *M*_f_ between the three materials. Besides, the larger the B content, the more significant the hardenability, and the lower the *M*_s_ and *M*_f_. The *M*_s_ and *M*_f_ of material 2# are about 11 and 5 °C lower than material 1#, respectively, while the *M*_s_ and *M*_f_ of material 3# are about 103 °C and 46 °C lower compared to material 1#, respectively.

**Figure 14:**
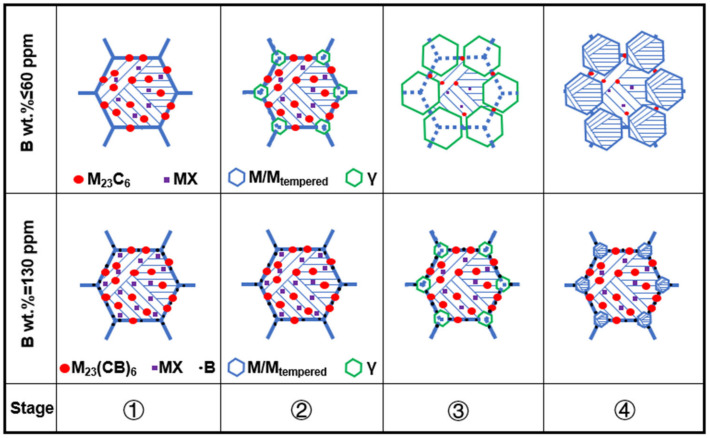
Effect of element B on ICHAZ microstructure evolution.① *T* < 818 °C, ② 818 < *T* < 913 °C, ③ 913 < *T* < 950 °C, ④ cooling from 950 °C to room temperature.

In ICHAZ, only the partial austenite formation and carbides redissolution could occur because *T*_p_ ranges between *A*_c1_ and *A*_c3_. In the materials without or with only a small amount of B, the stability of carbides was weak, and it was easy for them to be redissolved, thereby leading to the relative increase of alloy content in austenite. In this circumstance, the stability of the austenite was enhanced, resulting in a lower *M*_s_ and *M*_f_ than CGHAZ. Figure 14 shows a schematic diagram of the effect caused by element B on the microstructure evolution of ICHAZ. Due to the difference in B content between materials 1–3#, slight changes were occurring to carbides. The carbides in materials 1 and 2# were dominated by M_23_C_6_ and MX, while those in material 3# were dominated by M_23_(CB)_6_ and MX. Besides, B element segregation occurred at grain boundaries. In the first stage, when the welding thermal cycle temperature (*T*) was below 818 °C, the microstructure of materials 1–3# remained unchanged. In the second stage (818 < *T* < 913 °C), with *T* exceeding the *A*_c1_ of materials 1 and 2# but staying below that of material 3#, fine austenites started to come into form on the original austenite grain boundaries of materials 1# and 2#, and there were a small number of carbides redissolved, but no occurrence of austenite transformation in material 3#. In the third stage (913 < *T* < 950 °C), the austenite of materials 1 and 2# began to grow. Due to the low thermal stability of the carbides in materials 1# and 2#, the redissolution rate and amount of carbides began to increase. The number of carbides redissolved was high at this stage, while the austenite content was low compared with complete austenitizing. So, the alloy content in the austenite showed a sharp rise. Owing to *T* exceeding the *A*_c1_ of material 3#, fine austenites began to take shape on the original austenite grain boundaries. However, the carbides in material 3# exhibited high thermal stability, and boron atoms were segregated on the original austenite grain boundaries, with only a tiny amount of carbides redissolved. In the fourth stage (cooling from 950 °C to room temperature), the austenites in materials 1–3# were transformed into martensites. Due to the large number of carbides redissolved in materials 1 and 2# and the small number of austenites, the alloy content in austenites was significantly higher compared to complete austenite (CGHAZ), which would inevitably cause the *M*_s_ and *M*_f_ to further decrease. Besides, the *M*_s_ and *M*_f_ of material 1# decreased from the original 493 and 285 °C to 443 and 278 °C, while those of material 2# were reduced from 482 and 280 °C to 436 and 273 °C. However, only a very small amount of carbides redissolved into material 3# with high B content, while the alloy content in austenite was significantly lower compared to CGHAZ, as a result of which the *M*_s_ and *M*_f_ increased from 390 and 239 °C to 427 and 271 °C.

## 5. Conclusions

In this article, the effects of B content (0, 60, and 130 ppm), *T*_p,_ and *t*_8/5_ on the phase transformation, carbides, and microhardness of HAZ of G115 steel were also researched. The main conclusions were as follows:

(1) The microstructure of CGHAZ in G115 steel is coarse lath martensite. Besides, its FGHAZ is fine lath martensite, containing a small number of undissolved carbides, its ICHAZ is martensite and over-tempered martensite, and its SCHAZ is tempered martensite. B content (1#–0, 2#–60, 3#–130 ppm) and welding *t*_8/5_(25, 50, 150 s) made no difference to the microstructure of HAZ.
(2) The addition of element B affected the thermal stability of M_23_C_6_, thus resulting in a significant change to the distribution of undissolved carbides in the materials with varying B content given the same *T*_p_. The higher the B content, the larger the diameter and amount of undissolved carbides.
(3) The addition of element B can increase *A*_c1_ and *A*_c3_ for the materials. The *A*_c1_ and *A*_c3_ of material 3# increased by 95 and 108 °C, 69 and 77 °C compared to materials 1 and 2#, respectively. When the formation temperature range for FGHAZ was *A*_c3_–1250 °C, the residence time of material 3# in this temperature range was about 42.52% lower than material 1#, while the FGHAZ range and area could be significantly compressed.
(4) The content of B affected the pattern of change in *M*_s_ and *M*_f_ for materials 1–3# in ICHAZ. The alloy content in the austenite of materials 1 and 2# increased during the welding process, which was reduced by 50 and 7 °C, 46 and 7 °C compared to CGHAZ, respectively. The alloy content in the austenite of material 3# decreased by 37 and 32 °C compared to CGHAZ, respectively.

## Figures and Tables

**Table 1:** Chemical composition of G115 steel (wt.%).

Material	C	Si	Mn	Cr	Co	W	V	Nb	Cu	B	N	Fe
1#	0.071	0.32	0.53	8.96	3.01	2.73	0.19	0.067	0.79	0	0.007	Bal.
2#	0.069	0.32	0.53	9.02	3.02	2.74	0.20	0.066	0.78	0.006	0.008	Bal.
3#	0.076	0.18	0.45	8.83	2.99	3.11	0.19	0.042	0.85	0.013	0.008	Bal.

**Table 2:** Welding thermal stimulation schemes of G115 Steel.

Sub-Zone	Heating Rate *v/*(°C s^−1^)	Holding Temperature *T*_p_/°C	*t*_8/5_/s
CGHAZ	200	1350	25, 50, 150
FGHAZ	200	1150	25, 50, 150
ICHAZ	200	950	25, 50, 150
SCHAZ	200	770	25, 50, 150

**Table 3:** Composition structural formula of M_23_C_6_ carbide [10].

Material	Composition Structure of M_23_C_6_ Carbides
B wt.% = 0 ppm	(Fe_0__.242_Cr_0__.653_W_0__.049_Co_0.009_)_23_C_6_
B wt.% = 60 ppm	(Fe_0__.245_Cr_0__.644_W_0__.048_Co_0.008_)_23_(C_0__.926_B_0.074_)_6_
B wt.% = 140 ppm	(Fe_0__.244_Cr_0__.647_W_0__.048_Co_0.009_)_23_(C_0__.844_B_0.156_)_6_

## Data Availability

The data presented in this study are available upon request from the corresponding author.
