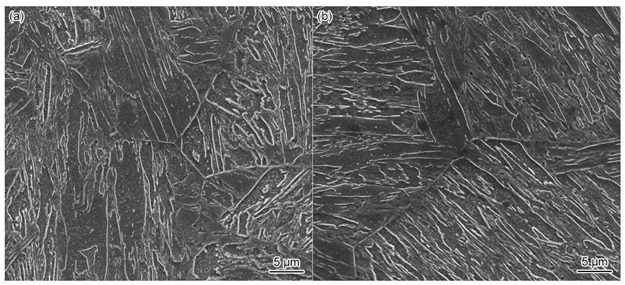# Correction: Chen et al. Evolution of Microstructure in Welding Heat-Affected Zone of G115 Steel with the Different Content of Boron. *Materials* 2022, *15*, 2053

**DOI:** 10.3390/ma17081731

**Published:** 2024-04-10

**Authors:** Zhongyi Chen, Dongxu Kou, Zhengzong Chen, Fan Yang, Yonglin Ma, Yiming Li

**Affiliations:** 1 School of Material and Metallurgy, Inner Mongolia University of Science and Technology, Baotou 014010, China; kdx2020023011@163.com (D.K.); malin@imust.cn (Y.M.); 2 Institute for Special Steels, China Iron and Steel Research Institute, Haidian, Beijing 100081, China; czz1223@126.com; 3 Inner Mongolia Shangdu Power Company, Xilin Gol League, Xilinhot 027200, China; yfan202202@163.com; 4 Key Laboratory of Advanced Metals and Materials, School of Materials and Metallurgy, Inner Mongolia University of Science and Technology, Baotou 014010, China; liyiming79@sina.com

In the original publication [1], there were two mistakes in Figure 4. Due to the authors’ negligence, Figure 4a,b was presented incorrectly in the original publication. The corrected Figure 4a,b appears below. The authors state that the scientific conclusions are unaffected. This correction was approved by the Academic Editor. The original publication has also been updated.